# Cancer chemoprevention and therapy using chinese herbal medicine

**DOI:** 10.1186/s12575-017-0066-1

**Published:** 2018-01-08

**Authors:** Lijing Jiao, Ling Bi, Yan Lu, Qin Wang, Yabin Gong, Jun Shi, Ling Xu

**Affiliations:** 1 Institute of Clinical Immunology, Yueyang Hospital of Integrated Traditional Chinese and Western Medicine, Shanghai University of Traditional Chinese Medicine, Shanghai, 200437 China; 2 Department of Oncology, Yueyang Hospital of Integrated Traditional Chinese and Western Medicine, Shanghai University of Traditional Chinese Medicine, No.110, Ganhe Road, Hongkou District, Shanghai, 200437 China

**Keywords:** Cancer, Chemoprevention, Treatment, Chinese herbal medicine

## Abstract

Traditional Chinese medicine (TCM) plays an indispensable role in cancer prevention and treatment. Chinese herbal medicine (CHM) is a key component of TCM and has been practiced for thousands of years. A number of naturally occurring products from Chinese herbs extracts exhibit strong inhibitory properties against carcinogenesis, including CHM single-herb extracts, CHM-derived active components, and CHM formulas (the polyherbal combinations), which regulate JAK/STAT, MAPK, and NF-ҡB pathways. The present review aims to report the cancer-preventive effect of CHM with evidence from cell-line, animal, epidemiological, and clinical experiments. We also present several issues that have yet to be resolved. In the future, cancer prevention by CHM will face unprecedented opportunities and challenges.

## Background

It is estimated that approximately 14.1 million new cancer cases and 8.2 million cancer deaths occurred in 2012 worldwide [1]. Although many significant advancements in cancer treatment have been applied clinically, the morbidity and mortality of cancer remain high. An important reason for this unsatisfactory situation is that less attention has been paid to cancer prevention than treatment. Cancer can be caused by a variety of factors and may develop over a long time period, even during treatment. Medications that interrupt or reverse precancerous changes would be appealing and cost-effective in the battle against cancer.

The molecular prevention of cancer can be defined as the use of natural or synthetic agents that interrupt the prime drivers, key derangements, or the context in which these drivers act and derangements occur, before invasion across the basement membrane [2]. Chemoprevention is one aspect of the molecular prevention of cancer and was first defined by Sporn in 1976 [3]. Chemoprevention has been defined as the use of specific natural or synthetic chemical agents to reverse, suppress or prevent carcinogenic development to a tumor, which involves natural drugs or micronutrients that inhibit cancer development either by blocking cancer initiation through DNA-damaging agents, or by arresting or reversing the progression of initiated cells, except for vaccines and therapeutic interventions against microbal related cancer by antimicrobial effects (e.g., *Helicobacter pylori* for gastric cancer) [4]. Plants and their effective ingredients account for a large proportion of natural agents that have been used for cancer prevention and treatment in traditional Chinese medicine (TCM) clinical practice for ages, and some naturally occurring products from Chinese herbal medicine (CHM) exert chemopreventive properties against carcinogenesis. Research regarding the anti-proliferative and cytotoxic effects of TCM is being pursued to develop evidence-based complementary and alternative medicine or drug discovery (Table 1), which indicates that TCM could be a potential approach for chemoprevention.

**Table 1:** Chinese Herbs in Chemoprevention Models

Chinese Drug name	Chemoprevention model in vitro	Traditional application	Potential Active Components
*Green tea*	DMBA or UVB-induced skin papillomas in CD-1 mice [5]	Traditional Chinese drinks	Epicatechin-3-gallate [83, 84]
	N-nitrosomethylbenzylamine (NMBzA) oesophageal tumor caused by in rats [6]		Epigallocatechin gallate [85, 86]
	N-nitrosodiethylamine(NDEA)-induced forestomach and lung cancer in A/J mice [7]		Epigallocatechin-3-gallate [87, 88]
	UVB-induced skin tumor in SKH-1 mice 8 [89]		
	NNK induced lung cancer in A/J mice [90]		
	N-Methyl-N-nitrosourea-induced colon carcinogenesis in F344 rats [91]		
	B[a]P-induced lung cancer in A/J mice [8]		
	NDEA-induced lung tumorigenesis in A/J mice [7]		
	Transgenic adenocarcinoma of the mouse prostate (TRAMP) model [92, 93]		
	NNK-induced lung cancer in A/J mice [94]		
	N-ethyl-N′-nitro-N-nitrosoguanidine (ENNG)-induced duodenal tumors in C57BL/6 J mice [95]		
	UVB-induced skin tumors [94]		
*Prunella vulgaris*	B[a]P-induced lung cancer in A/J mice [10]	Diabetics	Quercetin [96]
	B[a]P, 1,6-dinitropyrene and 3,9-dinitrofluoranthene [97]		Ursolic acid [98] Luteolin [99]
Rosmarinic acid *(Effective Components extracted from Salvia miltiorrhiza)*	DMBA-induced oral carcinogenesis in golden Syrian hamsters [12]	Coronary artery disease, gastric ulcer and tumor	NA
	DMBA-induced skin carcinogenesis in Swiss albino mice [13]		
Ginseng	B[a]P-induced lung cancer in A/J mice [8]	Chronic lung disease, antioxidant and tumor	20(S)-Protopanaxadiol [100] Ginsenoside-Rh2 [101, 102] Ginsenoside F2 [103] Ginsenoside-Rb1 [104]
	DMBA, urethane, and aflatoxin B1-induced Lung cancer in ICR newborn mice [19]		
	NTCU-induced lung SCC in Swiss mice [20]		
	DMBA-induced chromosomal aberra-tions and micronuclei [21]		
	TPA-induced skin tumor in ICR mice [105]		
*Scutellaria barbata*	DMBA and TPA-induced skin tumor in female (C57BL/6XC3H) F1 (B6C3F1) mice [22]	Tumor	Scutellaria barbata polysaccharide [106] Scutellarin [107] BZL101 (FDA IND# 59,521 [108]
*Curcumin*	Azoxymethane-induced rat colon carcinogenesis [24]	Antioxidant and	NA
*(extracted from Turmeric)*	4NQO-induced oral carcinogenesis [26] DMBA-induced oral carcinogenesis in hamsters [109]	anti-inflammatory	
*Honiokial*	male nude mice implanted with PC-3 cells [110]	Constipation and	NA
	UVB-induced skin cancer in mice [14]	abdominal distension	
	DMBA-initiated and TPA-promoted skin cancer in SKH-1 mice [15]		
	UVB-induced skin cancer in SKH-1 mice [16]		
	NTCU-induced lung SCC in Swiss mice [18]		
*Magnolol Magnolol* *(extracted from Mangnolia officinalis)*	UVB-induced skin cancer in mice [16, 111]	Constipation and abdominal distension	NA
*ATB* *(Formula contains Sophora tonkinensis, Polygonum bistorta, Prunella vulgaris, Sonchus brachyotus, Dictamnus dasycarpus, and Dioscorea bulbifera)*	N-butyl-(4-hydroxybutyl) nitrosamine (BBN)-induced precancerous lesions of the bladder in rats [27] B[a]P-induced in A/J mice harboring a dominant-negative p53 mutation and/or a heterozygous deletion of Ink4a/Arf [29] NTCU-induced lung SCC in Swiss mice [31] 4NQO-induced oral SCC in A/J mice [30] DMBA-induced buccal pouch carcinogenesis in hamsters [32]	Tumor	Sophocarpine [112, 113], Epigallocatechin,gallate [114],Quercetin [96], Ursolic acid [98],Luteolin [99] Obacunone [115] Psoralen [116]
Liu-Wei-Di-Huang Wan *(Formula contains Rehmannia glutinosa, Cornus officinalis Sieb., Common Yam Rhizome, Alisma orientalis, Tree Peony Bark, and Poria cocos)*	DMBA-induced lung cancer in mice Urethane-induced lung cancer in mice [34]	Osteoporosis, Alzheimer disease, hypertension, and diabetes	Catalpol [117, 118], Ursolic acid [98],Triterpenes [119], Paeonol [120, 121] Paeoniflorin [122] Pachymic acid [123, 124]

NA: not applicable

## Preclinical Studies in Cancer Chemoprevention

In the initial search for chemopreventive agents, animal models have been used extensively in the efficacy testing of potential chemopreventive agents. CHM has shown efficacy against multiple types of cancer (Table 2). The chemopreventive effects of green tea intake have been shown in many in vivo studies. For the 7,12-dimethylbenz(a)anthracene (DMBA) and 12-O-tetradecanoyl phorbol-13-acetate (TPA)-induced skin papillomas, partial tumor regression or >90% inhibition of tumor growth, and marked inhibition of tumor growth (46–89%) were observed by intervention with green tea [5]. The incidences of esophageal mucosa lesions and esophageal tumors were significantly lower in the tea-treated rats (16–59% and 42–67%, respectively), compared with the control group (100% and 90%) [6]. Moreover, aqueous extract of green tea inhibited carcinogen-induced lung tumorigenesis in mice by 63% [7]. Polyphenon E exhibited a significant reduction in both tumor multiplicity (by 46%) and tumor load (by 94%) [8], while Epigallocatechin-3-gallate (EGCG) and Poly E-EGCG did not significantly inhibit lung tumor multiplicity [9].

**Table 2:** Mechanism of Action of Herbal Mixtures

Name		Botanical Origin	Biological/Pharmacological Activity	Potential Active Components
Latin	Chinese Pinyin			
Single Herbs				
*Green tea*	Lv Cha	Nonfermented leaves of the plant *Camellia sinensis*	Apoptosis, cell cycle arrest, growth inhibition, antiangiogenesis, and inhibition of metastasis [87]	Epicatechin, Epigallocatechin, Epicatechin-3-gallate,Epigallocatechin-3-gallate [87],
*Panax ginseng C. A. Mey.*	Ren Shen	Fleshy roots	Apoptosis [125], cell cycle arrest, growth inhibition, antiangiogenesis, anti-tumor [125, 126]	EFLA400 [21]
Prunella vulgaris L.	Xia Ku Cao	Dried spikes of the Labiatae plant PV.	Immune modulatory [52, 127], antiestrogenic [128], antiestrogen receptor [51], anti-tumor [127]	Rosmarinic, ellagic and caffeic acids [129],Phenolic acids, flavonoids, coumarins, triterpense, volatile oil, polysaccharides [50, 127]
Scutellaria barbata D. Don	Ban Zhi Lian	Aqueous extract from Scutellaria barbata	Anti-inflammatory and anti-tumor [22]	BZL101 [22, 130]
*Magnolia officinalis*	Hou Pu	Root and stem bark of the oriental herb Magnolia officinalis	Anti-inflammatory [131], anti–gastric ulcer, anti-allergic, antibacterial, and antithrombotic properties [110],	Honokiol and magnolol [132, 133],rosmarinic acid
*Curcuma longa L.*	Jiang Huang	Root of the *Curcuma Longa*	Anti-inflammatory, antioxidant [134, 135]	Curcumin [23]α-pinene, β-pinene, γ-terpinene, p-cymene, cuminaldehyde, carvone, 1,8-cineole, β-carotene, β-sitosterol, caffeic acid,carvacrol, carvaol, geranial, kaempferol, limonene, p-coumaric acid, quercetin, tannin, thymol [135]
Formula				
Anti-tumor B (ATB), also known as Zeng Sheng Ping(增生平)	Zeng Sheng Ping Pian	Mixture composed of six plants: *Sophoratonkinensis, Polygonum bistorta, Prunella vulgaris, Sonchus brachyotus, Dictamnus dasycarpus, and Dioscorea bulbifera.*	Anti-tumor [29]	NA
BaoFei Decoction	Bao Fei Yin	*Clerodendrum bungei,* *Solanum nigrum L.,* *Platycodon grandiflorus,* *Glycyrrhiza uralensis Fisch,*	Anti-tumor [136, 137]	NA
Liu-Wei-Di-Huang Wan	Liu Wei Di Huang Wan	Mixture composed of Six plants: *Formula contains Rehmannia glutinosa, Cornus officinalis Sieb., Common Yam Rhizome, Alisma orientalis, Tree Peony Bark, and Poria cocos*	Immune modulatory, anti-tumor [34, 35]	NA

NA: not applicable

As an extract of *Prunella vulgaris L* (PV) with 60% ethanol, P-60 could be used to treat B[a]P-induced lung cancer and decrease the tumor multiplicity by 90.3% [10]. Rosmarinic acid (RA) is a caffeic acid-related compound abundant in PV [11], whose oral administration completely prevented tumor formation induced by DMBA in hamsters [12] and had potent anti-cancer, anti-lipid peroxidative, and apoptotic effects on DMBA-induced skin carcinogenesis [13].

Honokiol, a plant lignan isolated from bark and seed of the cones of *Magnolia officinalis*, has shown chemopreventive effects on chemically induced skin cancer development [14]. It has been reported to delay the formation of papillomas with a 27–55% reduction in tumor multiplicity in mouse skin initiated by DMBA and promoted by TPA [15]. α-santalol combined with honokiol and magnolol as pretreatment decreased tumor multiplicity (up to 75%) on skin cancer in SKH-1 mice [16]. In an A549 lung cancer xenograft model, the combination of honokiol with cisplatin reduced the tumor volume (3.59-fold), compared with cisplatin alone [17]. Honokiol also reduced the percentage of bronchial disease exhibiing abnormal histology (squamous cell carcinoma, SCC) (from 24.4 to 11.0%, *P* = 0.01) and protected normal bronchial histology (20.5% in the control group and 38.5% in the honokiol-treated group, *P* = 0.004) [18].

*Ginseng* is another well-studied herb that shows strong chemopreventive activities. In a lung adenoma model induced by 48 weeks of DMBA, it decreased the average diameter of the largest lung adenomas by 23% and the incidence of diffuse pulmonary infiltration by 63%. In the *Ginseng* treatment group sacrificed 56 weeks after birth (aflatoxin B1 combined with *Ginseng*), the incidence of lung adenoma (29%) and hepatoma (75%) was decreased [19]. Oral administration of aqueous extract of red *Ginseng* decreased tumor multiplicity by 36% and the tumor load by 70% [8]. *Korea White Ginseng* (KWG) significantly reduced the percentage of SCC to 9.1%, compared with 26.5% in the control group. KWG also significantly reduced the squamous cell lung tumor area to an average of 1.5%, compared with 9.4% in the control group [20].

EFLA400 is a standardized *Panax ginseng* extract containing a high titre of ginsenoside Rg3 (>3.0% *w*/w). Oral administration of EFLA400 at pre-, peri-, and post-initiational phases showed reductions in tumor incidence (71.41 ± 6.73%, 72.19 ± 4.54%, and 70.46 ± 0.38% at 1, 3, and 10 mg/kg body weight, respectively), compared with 100% tumor incidence in the control group [21].

*Scutellaria barbata D. Don* (Lamiaceae) (SB) is known in CHM as Ban-Zhi-Lian. It has been used as an anti-inflammatory and anti-tumor agent. During an 18-week study, mice treated with DMBA plus TPA developed 3.5 tumors per mouse with a 34% tumor incidence on average. The application of 5, 10, 100, and 200 mg of SB extracts together with TPA reduced the numberof skin tumors by 35%, 43%, 50%, and 55%, respectively, and the percentage of mice with tumors were lowered by 45%, 55%, 60%, and 65%, respectively [22].

Curcumin (Diferuloylmethane) is the most important component of the spice turmeric and is derived from the rhizome of the East Indian plant *Curcuma longa* [23]. Curcumin in the diet of male F344 rats was shown to decrease the incidence of azoxymethane (AOM)-induced colon cancer, from 81% to 47% [24]. The combination of tea and curcumin significantly decreased the visible oral tumor incidence from 92.3% (24/26) to 69.2% (18/26) and the SCC incidence from 76.9% (20/26) to 42.3% (11/26). The combination also decreased the number of visible tumors and tumor volume by 52.4% and 69.8% and decreased the number of SCCs, dysplasic lesions, and papillomas by 62.0%, 37.5%, and 48.7%, respectively. Curcumin decreased the number of visible tumors (by 39.6%), the tumor volume (by 61.3%), and the number of SCCs (by 51.3%). Only the combination treatment decreased the proliferation index in SCCs [25]. Another study found that oral administration of curcumin during the initiation and postinitiation phases, as well as hesperidin at the initiation stage, caused a significant reduction in the incidence of tongue carcinoma (41–91% reduction, *P* < 0.05), and the order of chemopreventive efficacy was curcumin > β-carotene > hesperidin. The incidence of oral preneoplasia in rats fed with these compounds was also decreased (*P *< 0.05) [26].

Anti-tumor B (ATB), also called Zeng-Sheng-Ping, is a Chinese herbal mixture composed of six plants that has shown an anticancer effect in mouse models of bladder cancer [27], lung cancer [28, 29], and oral cancer [30]. Preclinical studies have shown that ATB could reduce the incidence of N-butyl-(4-hydroxybutyl) nitrosamide (BBN)-induced bladder cancer by 90.7% [27]. ATB caused a significant reduction in lung tumor multiplicity and tumor load (40% and 70%, respectively) [31]. In an oral SCC model, ATB decreased the incidence and multiplicity by 59.19% and 64.81%, respectively [30]. Both the ATB n-butanol fraction and water fraction significantly reduced the tumor volume by 32.6% (*P* < 0.01) and 22.9% (*P *< 0.01) in DMBA-induced buccal pouch carcinogenesis in hamsters [32]. Anti-tumor B inhibited 4-nitroquinoline-1-oxide (4NQO)-induced oral cancer development by 65% [30]. In a mouse model of 4NQO-induced oro-esophageal cancer, ATB (10% in diet) also significantly reduced the incidence of tongue SCC from 55.2% (16/29) to 22.2% (6/27) (*P* < 0.05) and slightly reduced the incidence of esophageal SCC from 34.5% (10/29) to 22.2% (6/27) [33]. In B[a]P-induced mouse lung adenomas, ATB reverted 40% of gene expression changes to normal levels [31], and most of these ATB-modulated genes were involved in cell proliferation. ATB is a potential agent for human lung adenocarcinoma carrying common genetic alterations.

Liu-Wei-Di-Huang-Wan (LP) is an ancient Chinese prescription consisted of six herbs: *Rehmannia glutinosa, Cornus officinalis Sieb, Common Yam Rhizome, Alisma orientalis, Tree Peony Bark,* and *Poria cocos.* It could inhibit the incidence of theurethan-induced lung pulmonary adenomas by 50–56% [34, 35]. To our knowledge, no relevant studies on LP in chemoprevention have been published in the last 20 years.

These results suggest that CHM could be a potential chemopreventive agent for cancer. Moreover, the findings from the in vivo studies have shown that CHM can exert potent chemopreventive effects against many types of cancer.

## Mechanisms of Action

Considering the complicated factors of tumorigenesis, several pathways are believed to play an important role in chemoprevention. For example, the aberrant activation of intracellular signaling pathways confers malignant properties on cancer cells via the JAK/STAT and MAPK pathways [36, 37]. Chronic inflammation or tissue damage resulting in persistent inflammation promotes cell transformation through genetic damage or pro-inflammatory cytokines, thereby inducing chronic inflammation and tumorigenesis, which is activated by the NF-ҡB pathway [38]. Moreover, physiological cellular signaling mechanisms normally tightly regulate the ability of cells to gain access to and utilize nutrients, posing a fundamental barrier to transformation, which is abolished by the PI3K-Akt-mTOR pathway and then causes tumorigenesis [39].

Recent preclinical studies have improved our understanding of the mechanisms of CHM for chemoprevention (Table 2). In vitro studies, have demonstrated that green tea and EGCG could blocked carcinogenesis by affecting a wide range of signal transduction pathways: JAK/STAT [40], MAPK [41], PI3K/AKT [42], Wnt [43], NF-ҡB [44], Notch [45], and STAT3 [46]. The results demonstrated the beneficial effects of quercetin and EGCG on the suppression of the JAK/STAT cascade of CCA cells [40]. One study suggested that EGCG could suppress the proliferation anhe d induce tapoptosis of PANC-1 cells. Moreover, EGCG could upregulate PTEN expression and downregulate the expression of pAKT and p-mTOR to modulate the PI3K/AKT/mTOR signaling pathway [42]. EGCG exerts its cancer-preventive or anticancer activity against colon cancer cells by promoting the phosphorylation and proteasomal degradation of β-catenin through a mechanism independent of GSK-3βand PP2A [43]. The EGCG-induced apoptosis of HCCLM6 cells has been associated with a significant decrease in Bcl-2 and NF-kappaB expression. In addition, the expression of Bax, P53, caspase-9, and caspase-3 were increased, and Cytochrome C was released. These results suggest that EGCG inhibits the progression of cancer through cytocidal activity, and it is a potential therapeutic compound for hepatocellular carcinoma (HCC) [44]. EGCG has also been found to inhibit colorectal cancer by inhibiting HES1 and Notch2 [45]. Evidence shows that Polyphenon E(Poly E) treatment inhibits migration of MDA-MB231 breast cancer and human dermal microvascular endothelial (HMVEC) cells as well as the expression of VEGF and MMP9 through STAT3 [46]. Recent observations that β-catenin is upregulated in skin tumors suggests the possibility that the anti-skin carcinogenic effects of EGCG are mediated, at least in part, through its effects on β-catenin signaling. It was found that the EGCG treatment on the A431 and SCC13 human skin cancer cell lines resulted in reduced cell viability and increased cell death, and these cytotoxic effects were associated with the inactivation of β-catenin signaling [47]. EGCG inhibited the proliferation of Eca-109 and Te-1 cells in a time- and dose-dependent manner. Tumor cells were arrested in the G1 phase, and apoptosis was induced by ROS production and caspase-3 cleavage [48].

*Prunella vulgaris* (PV) extract and *Rosmarinic acid* (RA) also significantly eliminated ROS production and diminished IL-6 release to prevent UVB-caused DNA damage and oxidative stress to HaCaT keratinocytes [49]. RA inhibited TNF-α-induced ROS generation and NF-ҡB activation and enhanced TNF-α-induced apoptosis [50]. RA also suppressed the expression of MMP-9 by inhibiting NF-ҡB via the ERK1/2 signaling pathway as well as MMP-9 activity [51]. In addition, PV induced gene expression and the production of macrophage-related cytokines, such as TNF-α, IL-1b, and IL-6. PV stimulated macrophage activation via NF-ҡB transactivation and Mitogen-activated protein (MAP) kinase activation [52].

Multiple mechanisms have been implicated in the chemopreventive action of ginsenosides. One study showed that KWG functions as a chemopreventive agent through pathways involving AP-1, and KWG may partially depend on AP-1 for its chemopreventive function, possibly through the inhibition of JNK phosphorylation [20]. Ginsenoside Rg3, one of the major ingredients of heat-processed *Ginseng*, has been reported to inhibit the growth of various cancer cells. Rg3 induced apoptosis in MDA-MB-231 cells by blocking the NF-ҡB signaling pathway via the inactivation of ERK and Akt as well as the destabilization of mutant P53 [53].

BZL101, as an aqueous extract from SB, exhibits selective cytotoxicity through strong induction of ROS in tumor cells, leading to the hyperactivation of poly (ADP-ribose) polymerase, followed by a sustained decrease in the levels of NAD and the depletion of ATP [54]. Anti-tumor and anti-angiogenic activities of SB extracts in LoVo and human umbilical vein endothelial cells are partially mediated by the inhibition of Akt/protein kinase B. This inhibition was Akt kinase-specific, as it had no effect on PI3K, the upstream kinase of Akt, whereas the levels of phosphorylated Bad and FHKR, the two downstream targets of Akt, changed as the levels of Akt changed [55].

Curcumin has also been shown to exert significant growth inhibitory effects on pre-cancerous and carcinoma cell lines, such as epithelial breast cell lines MCF-10A, MCF-7, BT-474, SK-BR-3-h, and MDA-MB-231 [56], and lung cancer cell lines, such as A549, PC-9, H1975, and H1650 [23]. A number of studies have suggested that curcumin has the potential to target cancer stem cells through the regulation of self-renewal pathways (Wnt/beta-catenin, Notch, sonic hedgehog) and specific microRNAs involved in the acquisition of the epithelial-mesenchymal transition [57]. A recent study also demonstrated that curcumin and its analogues (PGV-0 and PGV-1) enhance the doxorubicin cytotoxicity to MCF-7 cells by inhibiting HER2 activity and activating NF-ƙB [58]. Other recent findings incidate that curcumin may subvert the TGF-β signaling to an alternative adipogenic differentiation program in addition to the previously established interference with the osteomimetic properties, thus inhibiting the bone metastatic processes in a chemopreventive as well as therapeutic setting [59].

A number of findings have suggested that honokiol targets multiple signaling pathways, including NF-ҡB, STAT3, epidermal growth factor receptor (EGFR), and mammalian target of rapamycin (m-TOR), which play an important role in cancer initiation and progression [60]. A recent study showed that honokiol inhibited lung SCC cells’ proliferation, arrested cells at the G1–S cell-cycle checkpoint, and induced apoptosis. By interfering with mitochondrial respiration, honokiol changed the redox status in the mitochondria, triggered apoptosis, and finally led to the inhibition of lung SCC [18].

Previous findings showed that ATB modulated the expression of genes on multiple signaling pathways, such as the Notch and Ras-MAPK pathways [29]. The number of BrdU-labeled positive cells in the oral pre-cancerous tissues was significantly decreased after treatment with ATB butanol or water fractions, which inhibited oral tumor cell growth and reduced the expression of MAPK. In addition, ATB promoted tumor cell apoptosis by increasing Caspase-3 expression but decreasing Bcl-2 protein production [32]. Cell proliferation, silver stained nucleolar organizer region (AgNOR), and proliferating cell nuclear antigen (PCNA)-labeling index were also significantly suppressed by ATB treatment [33]. The expression of EGFR and phosphorylated EGFR (Tyr1173) was also down-regulated by ATB [30].

## Pharmacokinetics Studies

Pharmacokinetic (PK) data are important for understanding the interactions between Chinese herbs and cancer prevention. Satisfactory PK information on Chinese herbs is not available due to the low quantity and quality of relevant studies. However, there have been some PK analyses of green tea [61], *Ginseng* [62]*,* and curcumin [63]. In a PK model for curcumin, the area under the curve for 10 and 12 g doses was estimated (mean +/− SE) to be 35.33 +/− 3.78 and 26.57 +/− 2.97 mug/mL x h, respectively, and C(max) was 2.30 +/− 0.26 and 1.73 +/− 0.19 mug/ml. The T (max) and T (1/2) were estimated to be 3.29 +/− 0.43 and 6.77 +/− 0.83 h. The ratio of glucuronide to sulfate was 1.92:1. The curcumin conjugates were present as either glucuronide or sulfate or mixed conjugates [63].

## Toxicity Studies

Safety is as important as efficiency for chemoprevention agents. Green tea and curcumin are appealing for their low or non-toxicity. No pathologic changes in the liver, lungs, kidneys, etc., were found by microscopic examination after the administration of liposomal honokiol or liposomal honokiol plus cis-Dichlorodiamineplatinum (DDP). No adverse consequences occurred in gross measures, such as weight loss, ruffling of fur, life span, behavior, or feeding [17]. No overt signs of the SB-induced toxicity were observed, as judged by visual inspection of skin, gross morphological examination of major organs, and changes in body weights [22].

Several studies have shown that CHM, e.g., ATB, may cause some degree of toxicity in animals and human beings. Experimental animal studies and epidemiological surveys have uncovered green tea polyphenols’ (GTPs) toxicity at high doses, presumably due to pro-oxidative properties. Recent studies have shown that unlike low and medium dosage, diets containing high doses (1%) of GTPs aggravated colitis and colon carcinogenesis, caused nephrotoxicity and hepatotoxicity in mice, and down-regulated expressions of anti-oxidant enzymes and molecular chaperones [64, 65]. In a phase I trial to find the maximum tolerated dose of GTE, the dose-limiting toxicities were tremors, cough, constipation, and headache, which were thought to be caused by caffeine in GTE [66]. Another phase II study showed that GTE was well tolerated, although higher doses (750 and 1000 mg/m2) increased insomnia/nervousness without grade 4 toxicity [67]. ATB was well tolerated in A/J mice with doses as high as 400 g/kg diet. By giving diets composed of AIN-76A with ATB at 800 g/kg diet, mice lost body weight (>20%) within the first 2 weeks. These results are consistent with the long history (>26 years) of its safety profile in clinical trials and useage as herbal supplements [29]. However, the oral administration of ATB tablets caused severe side effects, including hepatic damage [68, 69], diarrhea, nausea, and rash [70], which limited the long-term administration of ATB for humans.

## Cancer Chemoprevention Clinical Trials with Chinese Herbs

Cancer chemoprevention clinical trials are vital for guiding the use of CHM in cancer prevention. Several clinical trials have shown benefits of CHM in cancer chemoprevention (Table 3).

**Table 3:** Clinical Trials in Chemoprevention with Chinese Herbs

Reference	Chinese herbs	Tumor Types	Type of Study	Number.of Patients	Administration Methods	Result	Conclusion	Adverse Events
Li N et a.l, (1999) [71]	Green tea	Oral leukoplakia	RCT double-blind	Tx = 29 Ctr = 30	Tx: Tea 3 g/day/Tea capsule 760 mg q.i.d.	Response rate: 37.9% in treatment arm vs 10% in control arm	Results provide some direct evidence on the protective effects of tea on oral cancer.	NA
Ahn WS et al. (2003) [138]	Green tea	High-risk (HPV infected) cervical lesions	Pilot study	Tx = 51 Ctr = 39	Tx1: Poly E Ointment 200 mg twice weekly Tx2: Poly E capsules 200 mg orally daily Tx3: EGCG capsules 200 mg orally daily Tx4:Poly E Ointment +Poly E capsules Ctr: nontreated	Overall 69% (35/51) in treatment arm vs 10% (4/39) patients in nontreated control (*P* < 0.05)	Green tea extracts can be a potential therapy regimen for patients with HPV-infected cervical lesions.	Hematological and non-hematological toxicities as well as adverse side effects in patients treated locallyor systemically with poly E and EGCG were evaluated at 4-week intervals for 12 weeks.
Tsao AS et al. (2009) [67]	Green tea	High-risk oral premalignant lesions (OPLs)	Phase II RCT	Tx1 = 11 Tx2 = 11 Tx3 = 9 Ctr = 10	Tx1: GTE 500 mg/m^2^ Tx2: GTE 750 mg/m^2^ Tx3:1000 mg/m^2^ Ctr: placebo thrice daily for 12 weeks	Response rate: GTE arms (*n* = 28; 50%) vs placebo (*n* = 11; 18.2%; *P* = 0.09). Two higher-dose GTE arms 58.8% (750 and 1000 mg/m2), 36.4% (500 mg/m2), and 18.2% (placebo); *P* = 0.03	The result suggested a dose-response effect; GTE may suppress OPLs, in part through reducing angiogenic stimulus (stromal VEGF).	Higher doses increased insomnia/nervousness but produced no grade IV toxicity
Yun TK et al. (2010) [76]	Red Ginseng	Chronic atrophic gastritis	RCT double-blind	Tx = 325 Ctr = 318	Tx: red ginseng (1 g) per week Ctr: placebo for 3 years	Male red Ginseng group showed a relative cancer risk of 0.35 (95% CI, 0.13–0.96; *P* = 0.03) compared to the male placebo	Administration of red ginseng extract powder for 3 years exerted significant preventive effects on the incidence of non–organ-specific human cancers in males.	Many subjects complained of gastroin- testinal symptoms: 55.0% in the placebo group and 57.3% in the red Ginseng group(*P* > 0.05)
Rugo H et al. (2006) [130]	BZL101	Advanced breast cancer	Phase I study	*N* = 21	Tx: 350 ml per day	There were no grade III or IV adverse events (AEs).	BZL101 was safe and had a favorable toxicity profile.	Grade I and II AEs included: nausea (38%), diarrhea (24%), headache (19%) flatulence (14%), vomiting (10%), constipation (10%), and fatigue (10%).
Robert E et al. (2011)	Curcumin	Aberrant crypt foci (ACF) in smoker	Phase IIa	*N* = 41 Tx1 = 22 Tx2 = 19	Tx1:2 g Tx2: 4 g daily for 30 days	40% reduction in the ACF number occurred with the 4 g dose (*P* < 0.005); while ACF was not reduced in the 2 g group in plasma curcumin/conjugate levels pre-and post-treatment (5-fold increase; *P* = 0.009) in the 4 g group.	Curcumin was well tolerated at both 2 g and 4 g, and it can decrease the ACF number.	61% had grade- I /II toxicity, primarily gastrointestinal disturbances. The single grade-III toxicity was atypical chest pain.
Lin PZ et al. (1990) [70]	ATB	Precancerous lesions of the esophagus	RCT Placebo	*N* = 2523 Tx1 = 841 Tx2 = 841 Ctr = 841	Tx1: ATB 8 tablets q.d Tx2: retinamide 25 mg q.d (1–6 months) 50 mg q.d (7–12 months) 100 mg q.d (13 months) Ctr: placebo	3 and 5 years after, the incidence of esophageal cancer in the ATB group was reduced by 52.2% and 47.3%, respectively. (*P* < 0.05)	This method needs further trial and study in high risk areas of esophageal cancer. The reliability of the experimental results is critically discussed.	1.67% diarrhea 0.6% nausea, rash
Wang J et al. (2000) [139]	ATB	Esophageal epithelial hyperplasia	Single-blind RCT placebo	Tx = 300 Ctr = 149	Tx: ATB 8 tablets b.i.d Ctr: placebo 8 tablets b.i.d	64.3% (193/300) response rate in treatment arm vs 22.8% (34/139) in control arm (*P* < 0.05)	ATB is an effective drug in treatment of esophageal epithelialhyperplasia.	Adverse effects are mild and well tolerated by patients.
Sun Z et al. (2010) [33]	ATB	Esophageal SCC in human patients with dysplasia	RCT	*N* = 112 Tx = 59 Ctr = 53	TX: ATB 4 tablets, 3 times per day for 8–12 months Ctr: placebo	Reduced the size of oral lesion in 67.8% (40/59) patients,whereas the placebo was effective in 17% (9/53) patients (*P* < 0.01).	ATB could prevent human patients with oral leukoplakia.	Drug toxicity was not monitored

Tx: Treatment group; Ctr: Control group; RCT: Randomized, placebo-controlled trial; NA: not applicable

The treatment group by using green tea showed a 37.9% response rate after 6 months on human oral precancerous mucosa lesions, compared with the control arm. There were differences in the number and total volume of AgNOR and the proliferating index of PCNA in oral mucosa cell nuclei between the treated group and the control group [71]. A phase I study showed that a dose of 1.0 g/m(2) tid (equivalent to 7 to 8 Japanese cups [120 ml] of green tea three times daily) for at least 6 months is recommended for future studies [66]. A phase II study of GTE suggested that higher doses of GTE may improve short-term (12 weeks) oral premalignant lesions’ (OPLs) outcome [67]. Many epidemiologic studies have been conducted to investigate the association between tea consumption and cancer. One conducted on 396 head and neck cancer (HNC) cases and 413 controls indicated an inverse association between HNC risk and green tea consumption, which appeared to be modified by alcohol drinking status [72]. For patients with asymptomatic Rai stage 0 to II chronic lymphocytic leukemia, studies showed that twice daily EGCG (2000 mg per dose) administration resulted in a 31% sustained reduction of ≥20% in the absolute lymphocyte count, and 69% of patients with palpable adenopathy experienced a ≥ 50% reduction in the sum of the products of all lymph node areas, which could be correlated with reductions in lymphadenopathy (correlation co-efficient, 0.44; *P* = 0.02) [73, 74]. Another phase II trial is ongoing to study how green tea extract works in preventing breast cancer compared to a placebo in 1084 cases of postmenopausal women by evaluating its effects on breast cancer biomarkers, including mammographic density, plasma insulin-like growth factor 1, IGF binding protein 3, and estrone (ClinicalTrials.gov Identifier: NCT00917735).

CHM is very effective in the chemoprevention of upper aerodigestive tract tumors. ATB has a long history of safe usage in thousands of patients for more than 30 years. One clinical study showed an approximately 50% reduction in the cancerization rate of marked esophageal dysplasia with ATB [70]. In this clinical trial, more than 2500 cases of marked esophageal dysplasia were randomly divided into an ATB group and a placebo control group [70]. After 3 or 5 years of treatment, the progression of esophageal dysplasia to esophageal cancer was inhibited remarkably by 52.2% and 47.3%, respectively [70]. After 9 years, the inhibition rate was 42.1% [75]. Previous studies have suggested ATB to be a promising chemopreventive agent in smokers with bronchial dysplasia (Steve Lam, Professor, British Columbia Cancer Agency, Vancouver, Canada. sclam@interchange.ubc.ca; personal communication). In a pilot study of ATB in smokers with bronchial dysplasia, 20 current and former smokers with a smoking history of approximately 30 pack-years and one or more sites of bronchial dysplasia identified by fluorescence bronchoscopy-directed bronchial biopsies were treated with ATB for 6 months. Using combined histopathology and nuclear morphometry as the primary end point, site-specific analysis showed a complete regression rate of 64% in the ATB group and 26% in the placebo group (*P* = 0.002). The corresponding progressive disease rates were 9% and 12% respectively. In a randomized clinical trial on 112 patients with oral leukoplakia, ATB (4 tablets, 3 times per day for 8–12 months) reduced the size of oral lesion in 67.8% (40/59) patients, whereas the placebo was effective in 17% (9/53) patients (*P* < 0.01) [33].

In a clinical trial on chronic atrophic gastritis patients, the administration of red ginseng extract powder for 3 years exerted significant preventive effects on the incidence of non–organ-specific human cancers in males [76]. The maximum dose of BZL101 (40 g/day) was safe, well tolerated, and showed promising anticancer activity in heavily pretreated population of women with metastatic breast cancer [77]. Curcumin is still lacking evidence of chemopreventive activity in humans. All 5 patients received more than 6 months of curcumin and quercetin, and the mean percent of decrease in the number and size of polyps from baseline was 60.4% (*P* < 0.05) and 50.9% (*P *< 0.05), respectively. Minimal adverse effects and no laboratory abnormalities were noted [78]. A phase II chemoprevention study of curcumin was conducted to examine the effect of oral curcumin on various putative biomarkers of colonic tumorigenesis in smokers and found that curcumin can decrease the aberrant crypt foci number [79].

## Perspectives and Conclusions

Cancer prevention by Chinese herbs is supported by studies from animal, cell culture, epidemiological, and clinical trials. However, Cancer prevention by Chinese herbs still a long way from clinical translation. More efforts are required for CHM standardization to ensure reproducibility, and the compatibility of different active compounds should be clarified, especially for the Chinese compound formula. In addition, with a more in-depth understanding of cross-reactivities and unintended consequences between CHM and other treatment, we could make better use of CHM in chemoprevention and treatment. Meanwhile, the increasing use of Chinese herbs around the world requires more scientific evidence for their putative harmlessness. The art of herbal medicine is to dissect pharmacologically and therapeutically valuable herbal drugs from harmful and toxic ones, and to develop combinations of medicinal plants as safe and efficient herbal remedies [80]. At this moment, the best way to ascertain the mechanism of chemopreventive agents on a molecular level is by using relevant biomarkers. Molecular markers can help in effectively determining the biological activity in a chemopreventive setting [81]. One review found that although 51 studies with more than 1.6 million participants have been done on cancer prevention, there is still limited evidence that green tea could reduce the incidence of liver cancer. The evidence for esophageal, gastric, colon, rectum, and pancreatic cancer remains conflicting [82]. Therefore, high methodological quality for clinical research is the key for clinical research to ascertain the cancer-preventive effect of Chinese herbs for cancer chemoprevention. Further research is expected to be done on developing agents with lower toxicity and higher efficacy for specific biomarkers and pathways, and targeting these therapies to individuals with specific genetic signatures should help to increase the utility of CHM in chemoprevention and treatment.

## Abbreviations

4NQO: 4-nitroquinoline-1-oxide
AgNOR: Silver stained nucleolar organizer region
AOM: Azoxymethane
AP-1: Activator protein-1
ATB: Anti-tumor B
BBN: N-butyl-(4-hydroxybutyl) nitrosamide
CH: Chinese herbs
CHM: Chinese herbal medicine
DDP: Cis-Dichlorodiamineplatinum
DMBA: 7,12-dimethylbenz(a)anthracene
EGCG: Epigallocatechin-3-gallate
EGFR: Epidermal growth factor receptor
ENNG: N-ethyl-N'-nitro-N-nitrosoguanidin
GSK-3ß: Glycogen synthase kinase-3ß
GTE: Green tea extract
GTPs: Green tea polyphenols
HCC: Hepatocellular carcinoma
HMVEC: Human dermal microvascular endothelial cell
HNC: Head and neck cancer
JAK/STAT: Janus kinase/ signal transducer and activator of transcription
KWG: Korea white ginseng
LP: Liu-wei-di-huang Wan
MAP: Mitogen-activated protein
MAPK: Mitogen-activated protein kinase
MMP9: Matrix metalloproteinase 9
NDEA: N-nitrosodiethylamine
NMBzA: N-nitrosomethylbenzylamine
NNK: 4-(methylnitro -samino)-1-(3-pyridyl)-1-butanone
NTCU: N-nitroso-trischloro-ethylurea
OPL: Oral premalignant lesions
PCNA: Proliferating cell nuclear antigen
PI3K/AKT: Phosphatidylin-ositol3-kinase / protein kinase B
PK: Pharmacokinetic
Poly E: Polyphenon E
PP2A: Phospho-protein phosphatease 2A
PTEN: Phosphatase and tensin homolog deleted on chromosome ten
PV: Prunella vulgaris
RA: Rosmarinic acid
ROS: Reactive oxygen species
SB: Scutellaria barbata
SCC: Squamous cell carcinoma
TCM: Traditional chinese medicine
TNF-a: Tumor necrosis factor-a
TPA: 12-O-tetradecanoyl phorbol-13-acetate
VEGF: Vascular endothelial growth factor
